# Long Noncoding RNA *FENDRR* Inhibits Lung Fibroblast Proliferation via a Reduction of β-Catenin

**DOI:** 10.3390/ijms22168536

**Published:** 2021-08-09

**Authors:** Lakmini Kumari Senavirathna, Yurong Liang, Chaoqun Huang, Xiaoyun Yang, Gayan Bamunuarachchi, Dao Xu, Quanjin Dang, Pulavendran Sivasami, Kishore Vaddadi, Maria Cristina Munteanu, Sankha Hewawasam, Paul Cheresh, David W. Kamp, Lin Liu

**Affiliations:** 1 Oklahoma Center for Respiratory and Infectious Diseases, Oklahoma State University, Stillwater, OK 74078, USA; lakmini.senavirathna@okstate.edu (L.K.S.); yurongl@okstate.edu (Y.L.); chaoqh@okstate.edu (C.H.); yangxiaoyun@gird.cn (X.Y.); gayan@wustl.edu (G.B.); daoxu@okstate.edu (D.X.); quanjin.dang@okstate.edu (Q.D.); pulaven@okstate.edu (P.S.); kvaddad@okstate.edu (K.V.); cristina.munteanu@okstate.edu (M.C.M.); shewawa@okstate.edu (S.H.); 2 Lundberg-Kienlen Lung Biology and Toxicology Laboratory, Department of Physiological Sciences, Oklahoma State University, Stillwater, OK 74078, USA; 3 Division of Pulmonary & Critical Care Medicine, Department of Medicine, Feinberg School of Medicine, Northwestern University, Chicago, IL 60611, USA; p-cheresh@northwestern.edu (P.C.); d-kamp@northwestern.edu (D.W.K.); 4 Department of Medicine, Division of Pulmonary & Critical Care Medicine, Jesse Brown VA Medical Center, Chicago, IL 60612, USA

**Keywords:** *FENDRR*, SRSF9, mTOR signaling, β-catenin

## Abstract

Idiopathic Pulmonary Fibrosis (IPF) is a chronic, progressive, and usually lethal lung disease and it has been widely accepted that fibroblast proliferation is one of the key characteristics of IPF. Long noncoding RNAs (lncRNAs) play vital roles in the pathogenesis of many diseases. In this study, we investigated the role of lncRNA *FENDRR* on fibroblast proliferation. Human lung fibroblasts stably overexpressing *FENDRR* showed a reduced cell proliferation compared to those expressing the control vector. On the other hand, *FENDRR* silencing increased fibroblast proliferation. *FENDRR* bound serine-arginine rich splicing factor 9 (SRSF9) and inhibited the phosphorylation of p70 ribosomal S6 kinase 1 (PS6K), a downstream protein of the mammalian target of rapamycin (mTOR) signaling. Silencing SRSF9 reduced fibroblast proliferation. *FENDRR* reduced β-catenin protein, but not mRNA levels. The reduction of β-catenin protein levels in lung fibroblasts by gene silencing or chemical inhibitor decreased fibroblast proliferation. Adenovirus-mediated *FENDRR* transfer to the lungs of mice reduced asbestos-induced fibrotic lesions and collagen deposition. RNA sequencing of lung tissues identified 7 cell proliferation-related genes that were up-regulated by asbestos but reversed by *FENDRR*. In conclusion, FENDRR inhibits fibroblast proliferation and functions as an anti-fibrotic lncRNA.

## 1. Introduction

Idiopathic Pulmonary Fibrosis (IPF) is a chronic and progressive scarring lung disease categorized under idiopathic interstitial pneumonia [1,2]. Lung fibroblast proliferation is a main characteristic of IPF as it results in extracellular matrix deposition, followed by damaging the lung parenchyma. The etiology of IPF is still unknown. However, it is believed that repeated micro-injuries to lung epithelium lead to aberrant proliferation and activation of lung fibroblasts, the failure to properly regulate the repair process, and the development of fibrosis [3,4].

Long noncoding RNAs (lncRNAs) have diverse cellular functions [5,6,7,8,9]. Several studies have shown that the involvement of lncRNAs in pulmonary fibrosis [10,11,12,13,14]. Our previous study has shown that FOXF1 Adjacent Non-Coding Developmental Regulatory RNA (*FENDRR*) has an anti-fibrotic activity by inhibiting the fibroblast activation and reducing the bleomycin-induced lung fibrosis in mice [15]. We have also demonstrated that *FENDRR* enhances the polarization of M1 macrophages via the STAT1 signaling [16]. *FENDRR* also has anti-proliferative effects in many cancer cells and has a role in tumor immunogenicity [17,18,19,20,21,22]. However, whether and how *FENDRR* regulates fibroblast proliferation in IPF is unknown. Based on our previous studies, we found that *FENDRR* binds with serine-arginine (SR) rich splicing factor 9 (SRSF9) [15] which is known to regulate mammalian target of rapamycin (mTOR) signaling [23].

Several signaling pathways are activated in IPF [24]. Among them, mammalian targets of rapamycin (mTOR) and Wnt/β-catenin signaling pathways play important roles in fibroblast proliferation [25,26]. β-catenin is known to promote cell proliferation in many cells [25,27,28,29]. One study has shown that tumor growth is promoted by β-catenin protein synthesis, which is enhanced by serine-arginine (SR) rich splicing factor 1 and 9 (SRSF1 and SRSF9) [23]. The cytoplasmic functions of SRSFs include activating mTOR signaling [30,31] and promoting protein translation [32]. SRSFs also act as oncogenic proteins to promote cell proliferation [23,30,33,34,35,36].

In this study, we hypothesize that the binding of *FENDRR* with SRSF9 negatively impacts mTOR signaling and cell proliferation. We found that *FENDRR* negatively affects fibroblast proliferation. We also showed that *FENDRR*, bound with SRSF9, inhibited the mTOR signaling pathway, and reducing β-catenin protein levels. Additionally, *FENDRR* reduced asbestos-induced lung fibrosis and collagen deposition in mice.

## 2. Results

### 2.1. FENDRR Inhibits Fibroblast Proliferation

**Figure 1:**
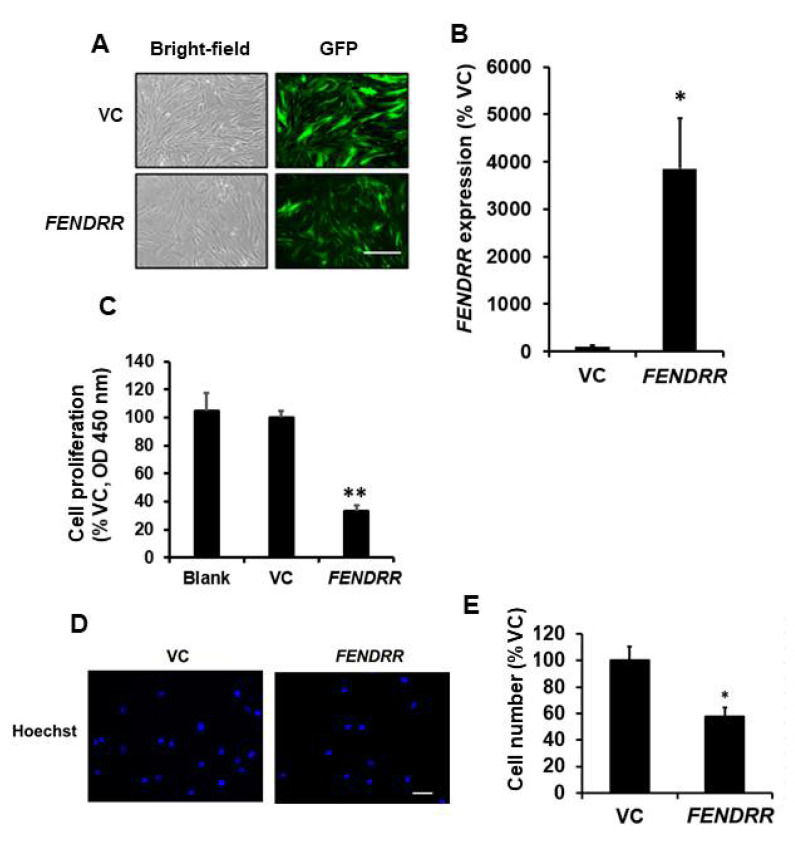
*FENDRR* has a negative effect on lung fibroblast proliferation. (**A**) Bright-field and GFP images of LL29 stable cells expressing GFP-*FENDRR (FENDRR)* and GFP vector control (VC). Scale bar: 100 µm. (**B**) Real-time PCR determination of *FENDRR* expression levels in the stable cells. *FENDRR* expression was normalized to β-actin and expressed as %VC. (**C**) Cell proliferation was determined by the BrdU assay in blank cells, VC, and *FENDRR* expressing stable cells after 6 days of culture. The results were expressed as %VC. (**D**) Nuclei staining of VC and *FENDRR* stable cells after 6 days of culture. Scale bar: 100 µm. (**E**) Cell counts of VC and *FENDRR* stable cells. In each experiment, 5 random fields were imaged, numbers of nuclei were counted, and cell counts were averaged. Cell counts were expressed as %VC. The cell counts for VC and *FENDRR* were 19.7 ± 2.0 and 11.3 ± 0.8 per field. Values represent the means ± SE. *n* = 3 independent experiments. * *p* < 0.05, ** *p* < 0.01 vs. VC. Student’s *t*-test for (**B**,**E**) and one-way ANOVA and Tukey’s multiple comparison for (**C**).

Since *FENDRR* expression is lower in lung fibroblasts from fibrotic lungs compared to those from normal lungs [15], IPF fibroblasts-LL29 were selected for overexpressing *FENDRR*. To examine the effects of *FENDRR* on fibroblast proliferation, we established a lung LL29 fibroblast line stably expressing GFP-*FENDRR* or GFP vector control (VC). GFP was observed in both GFP-*FENDRR* and GFP-VC lines (Figure 1A). The expression level of *FENDRR* in the GFP-*FENDRR* line was 39 ± 11 folds over the control cells (Figure 1B). The BrdU assay showed that *FENDRR* inhibited fibroblast proliferation by 68 ± 3% (Figure 1C). Cell counting revealed a 42 ± 7% reduction in the cell numbers in *FENDRR* overexpressing cells (Figure 1D,E).

### 2.2. Silencing of FENDRR Increases Fibroblast Proliferation

**Figure 2:**
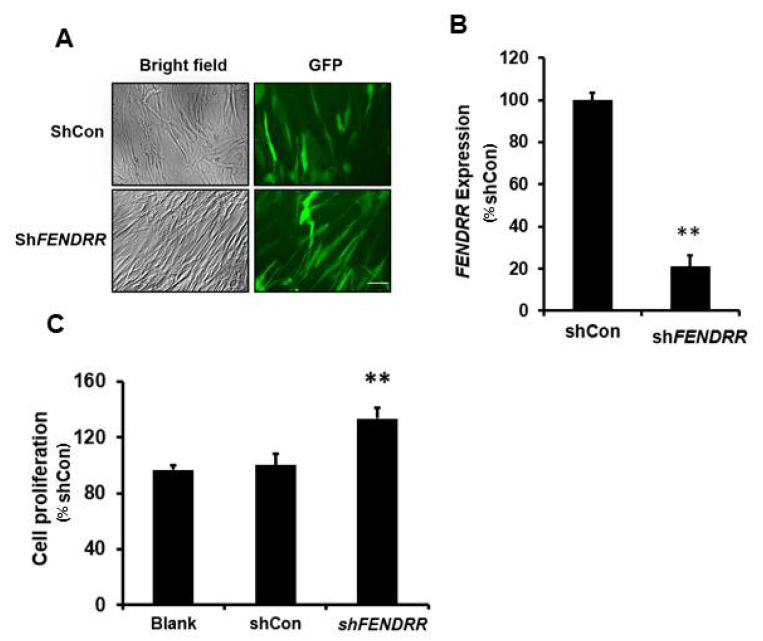
*FENDRR* silencing increases lung fibroblast proliferation. (**A**) Bright-field and GFP images of HPF cells infected with *FENDRR* shRNA (sh*FENDRR*) or shRNA control (shCon) lentiviruses (MOI 100 for 24 h). Scale bar: 100 µm. (**B**) Real-time PCR determination of *FENDRR* expression in HPF cells infected with sh*FENDRR* or shCon. *FENDRR* expression was normalized to β-actin and expressed as %shCon. (**C**) Cell proliferation was determined by the BrdU assay in blank cells and the cells infected with shCon and sh*FENDRR* after 6 days of culture. The results were expressed as %shCon. Values represent the means ± SE. *n* = 3 independent experiments. ** *p* < 0.01 vs. shCon. Student’s *t*-test for (**B**) and one-way ANOVA and Tukey’s multiple comparison for (**C**).

As mentioned above, *FENDRR* expression is lower in fibrotic lungs compared to normal lungs, and normal pulmonary fibroblasts were selected for *FENDRR* silencing. To determine whether silencing of endogenous *FENDRR* affects fibroblast proliferation, normal human pulmonary fibroblasts (HPFs) were infected with a lentivirus expressing a *FENDRR* shRNA construct with a GFP marker. GFP was observed in both shCon- and sh*FENDRR*-infected cells (Figure 2A). Real-time PCR analysis showed that the *FENDRR* expression level was reduced by 79 ± 5% (Figure 2B). The BrdU assay performed after 6 days of lentiviral infection showed that silencing of *FENDRR* increased fibroblast proliferation by 34 ± 8% (Figure 2C). These data further confirm that *FENDRR* is an inhibitory factor of lung fibroblast proliferation.

### 2.3. FENDRR Binds SRSF9 and Inhibits the Phosphorylation of PS6K

**Figure 3:**
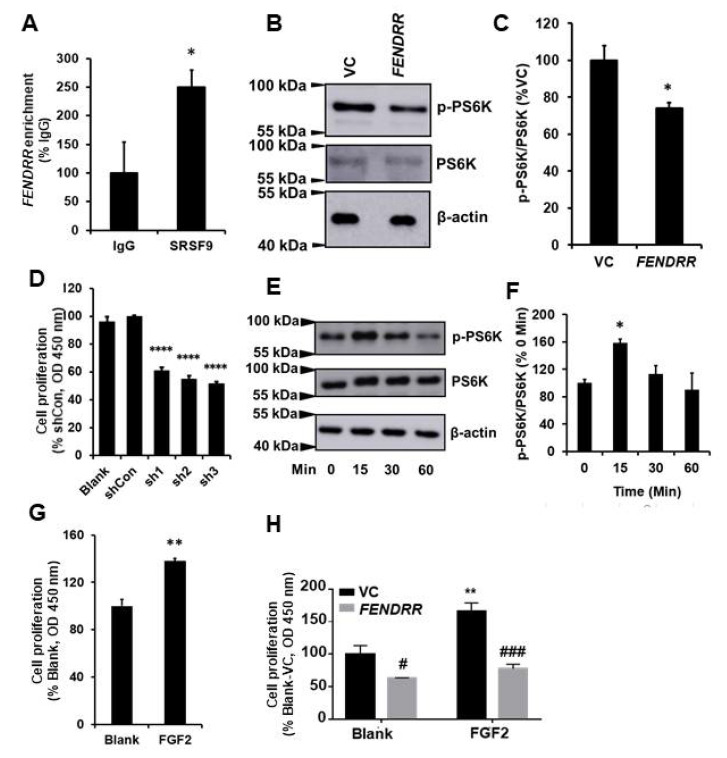
*FENDRR* reduces PS6K phosphorylation by binding SRSF9. (**A**) RNA immunoprecipitation showing the interaction of *FENDRR* with SRSF9 in LL29 cells. *FENDRR* enrichment was expressed as %IgG. (**B**) Western blot showing that *FENDRR* inhibits the phosphorylation of PS6K in LL29 stable cells expressing *FENDRR* or vector control (VC). (**C**) Quantitative analysis of protein levels of phosphorylated PS6K (p-PS6K). Values were normalized to total PS6K and then expressed as %VC. (**D**) Cell proliferation was determined by the BrdU assay at 6 days in LL29 cells infected with a lentivirus expressing a *SRSF9* shRNA (MOI of 100 for 24 h). Values were expressed as %shCon. (**E**) Western blot showing the phosphorylation of PS6K at different times points (0, 15, 30, and 60 min) in LL29 cells treated with FGF2 (50 ng/mL). (**F**) Quantitative analysis of the phosphorylated PS6K and total PS6K protein levels from (**E**). Values were normalized to total PS6K and then expressed as % 0 Min. (**G**) Fibroblast proliferation in LL29 cells treated with FGF2 (50 ng/mL) for 3 days as determined by the BrdU assay. Values were expressed as %Blank. (**H**) Fibroblast proliferation in LL29 stable cells expressing vector control (VC) or *FENDRR* treated with FGF2 (80 ng/mL) for 3 days was determined by the BrdU assay. Values were expressed as %VC of Blank. Values represent the means ± SE. *n* = 3 independent experiments. * *p* < 0.05 vs. IgG, VC, or 0 min, ** *p* < 0.01 vs. Blank, **** *p* < 0.0001 vs. shCon, ^#^ *p* < 0.05 and ^###^ *p* < 0.001 vs. VC. Student’s *t*-test for (**A**,**C**,**G**); one-way ANOVA and Tukey’s multiple comparison for (**D**,**F**); and two-way ANOVA and Tukey’s multiple comparison for (**H**).

RNA pulldown and mass spectroscopy analysis identified SRSF9 as one of the binding partners for *FENDRR* [15]. To confirm this result, an RNA immunoprecipitation assay was performed in LL29 cells. SRSF9 proteins were pulled down with anti-SRSF9 antibodies. RNAs bound with SRSF9 proteins were isolated, and *FENDRR* was determined by real-time PCR. Our results showed that SRSF9 proteins bound endogenous *FENDRR* in LL29 cells with a 2.4 ± 0.30-fold enrichment compared to the IgG control (Figure 3A).

The phosphorylation of PS6K protein, a downstream molecule of mTOR signaling, can be enhanced by SRSF9 [23]. The binding of *FENDRR* with SRSF9 may affect the PS6K phosphorylation. To test this possibility, we performed Western blotting on cell lysates extracted from *FENDRR* stable cells using antibodies against phosphorylated PS6K. The results showed that *FENDRR* reduced the phosphorylation of PS6K protein (Figure 3B,C). However, whether this effect is due to the binding of *FENDRR* to SRSF9 needs to be further confirmed via competition experiments.

SRSF9 has been reported as an oncogenic protein in several cancer cells [23,33,34]. To evaluate the contribution of SRSF9 to fibroblast proliferation, we silenced SRSF9 using a lentiviral shRNA vector in LL29 cells. The 3 shRNAs showed a similar knockdown efficiency (65–68%) (Figure S1A,B). The silencing of SRSF9 by all of the 3 shRNAs reduced fibroblast proliferation by 39–48% (Figure 3D).

We further examined the effects of activating mTOR signaling using FGF2 on fibroblast proliferation. FGF2 increased the phosphorylation of PS6K in LL29 cells at 15 min (Figure 3E,F). The treatment of LL29 cells with FGF2 for 3 days increased cell proliferation by 38 ± 2% as determined by the BrdU assay (Figure 3G). Furthermore, *FENDRR* reduced cell proliferation of both basal and FGF2-mediated cell proliferation (Figure 3H).

### 2.4. FENDRR Reduces β-Catenin Protein Level

**Figure 4:**
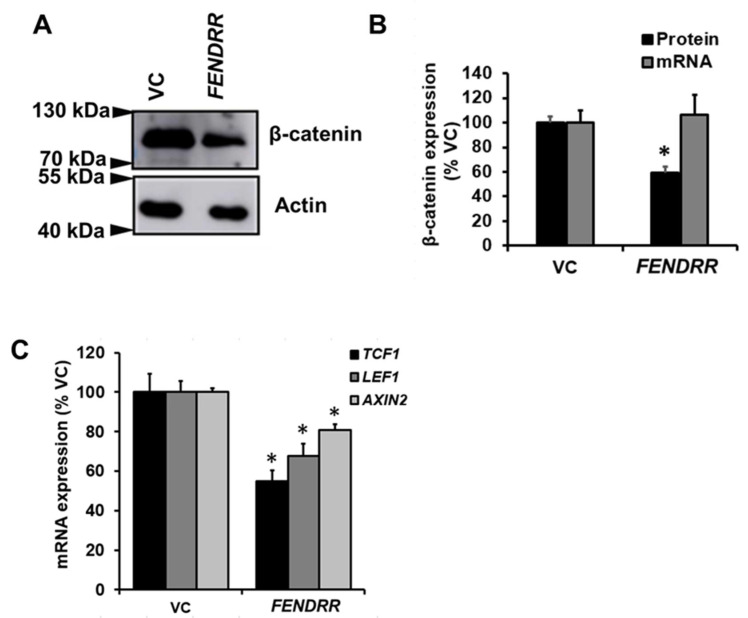
*FENDRR* reduces β-catenin protein level. (**A**) Western blot showing β-catenin protein levels in *FENDRR* overexpressing or vector control (VC) stable cells after a 6-day culture. (**B**) Quantification of β-catenin mRNA and protein expression. β-catenin mRNA expression was determined by real-time PCR. β-actin was used as the reference gene. Values were expressed as %VC. (**C**) mRNA expression of β-catenin target genes, *TCF1, LEF1*, and *AXIN2* in *FENDRR* and VC stable cells. mRNA expressions were determined by real-time PCR using β-actin as the reference gene and expressed as %VC. Values represent the means ± SE. *n* = 3 independent experiments. * *p* < 0.05 vs. VC. Student’s *t*-test for (**B**,**C**).

Since β-catenin promotes fibroblast proliferation [25] and overexpression of SRSF9 increases β-catenin protein levels in HEK293T [23], we wondered whether *FENDRR* regulates β-catenin expression in lung fibroblasts. Western blot analysis showed that stable cells expressing *FENDRR* had a 41 ± 5% reduction in β-catenin protein levels compared to control cells (Figure 4A,B). However, β-catenin mRNA was not changed (Figure 4B). The mRNA expression of β-catenin target genes, *TCF1, LEF1*, and *AXIN2* was reduced by *FENDRR* overexpression (Figure 4C).

### 2.5. Silencing and Overexpression of β-Catenin Reduces or Increases Fibroblast Proliferation

**Figure 5:**
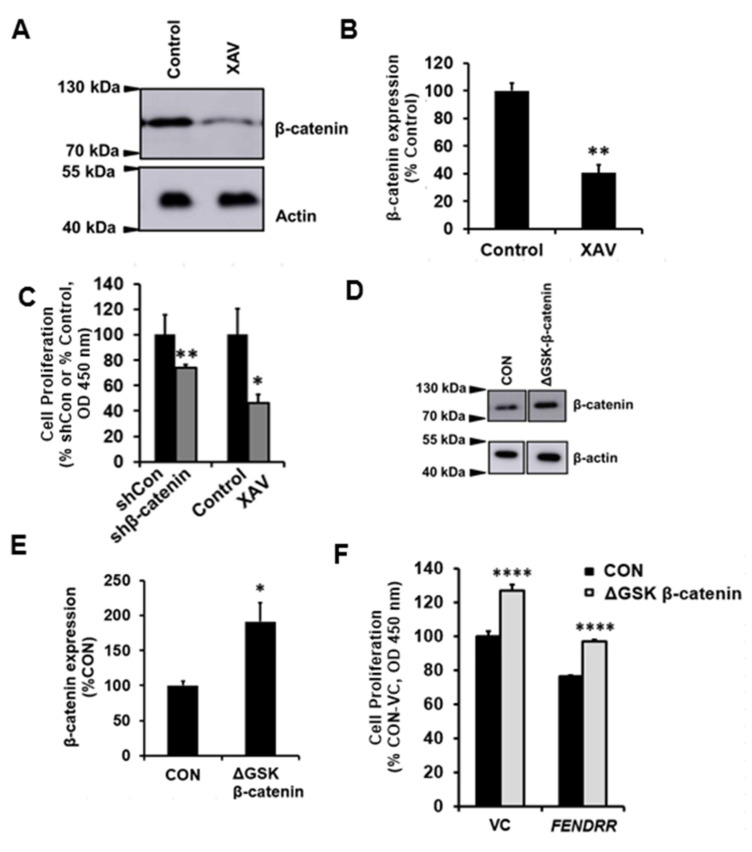
β-catenin contributes to fibroblast proliferation. (**A**) XVA939 (10 µM)-mediated reduction of β-catenin protein levels in LL29 cells. The control is 0.1% DMSO. (**B**) Quantitative analysis of β-catenin protein levels in LL29 cells treated with XAV939. The results were expressed as %VC. (**C**) BrdU assay of cell proliferation in LL29 cells infected with adenovirus containing β-catenin shRNA constructs or treated with XVA939 for 6 days. The results were expressed as %shCon or %Control. (**D**) Western blot showing β-catenin levels in LL29 cells after transfection (nucleofection) of ΔGSK-β-catenin overexpressing plasmid or its control plasmid (CON). (**E**) Quantitative analysis of β-catenin protein levels in D. The results were normalized to β-actin and expressed as %CON. (**F**) The BrdU assay was performed to determine cell proliferation in LL29 stable cells expressing vector control (VC) or *FENDRR* at day 6 after transfecting ΔGSK-β-catenin overexpressing plasmid or its control plasmid (CON). The results were expressed as %VC of CON. Values represent the means ± SE. *n* = 3 independent experiments. * *p* < 0.05, ** *p* < 0.01, **** *p* < 0.0001 vs. shCon, Control or VC. Student’s *t*-test for (**B**,**C**,**E**). Two-way ANOVA and Tukey’s multiple comparison for (**F**).

To mimic the *FENDRR*-mediated reduction of β-catenin, we reduced β-catenin levels in LL29 cells using adenoviral β-catenin shRNA and a chemical inhibitor and examined the effects of such treatments on fibroblast proliferation. Adenovirus-mediated β-catenin silencing reduced the β-catenin protein level by 49 ± 7% and mRNA level by 70 ± 3% (Figure S2A,B). A chemical inhibitor, XAV939 reduced the β-catenin protein level by 60 ± 6% (Figure 5A,B). β-catenin silencing and chemical inhibition reduced fibroblast proliferation by 26 ± 3% and 54 ± 7%, respectively (Figure 5C).

We then determined the effects of overexpressing β-catenin on the proliferation of vector control and *FENDRR* stable cells. We overexpressed β-catenin through transfecting ΔGSK β-catenin (a constitutively active form of β-catenin) [37] overexpressing plasmid using nucleofection technique. The overexpression increased the β-catenin protein level by 91.18 ± 27.08% (Figure 5E,F). The overexpression of β-catenin also increased the cell proliferation of both vector control and *FENDRR* overexpressing cells (Figure 5F).

### 2.6. FENDRR Attenuates Asbestos-Induced Pulmonary Fibrosis in Mice

**Figure 6:**
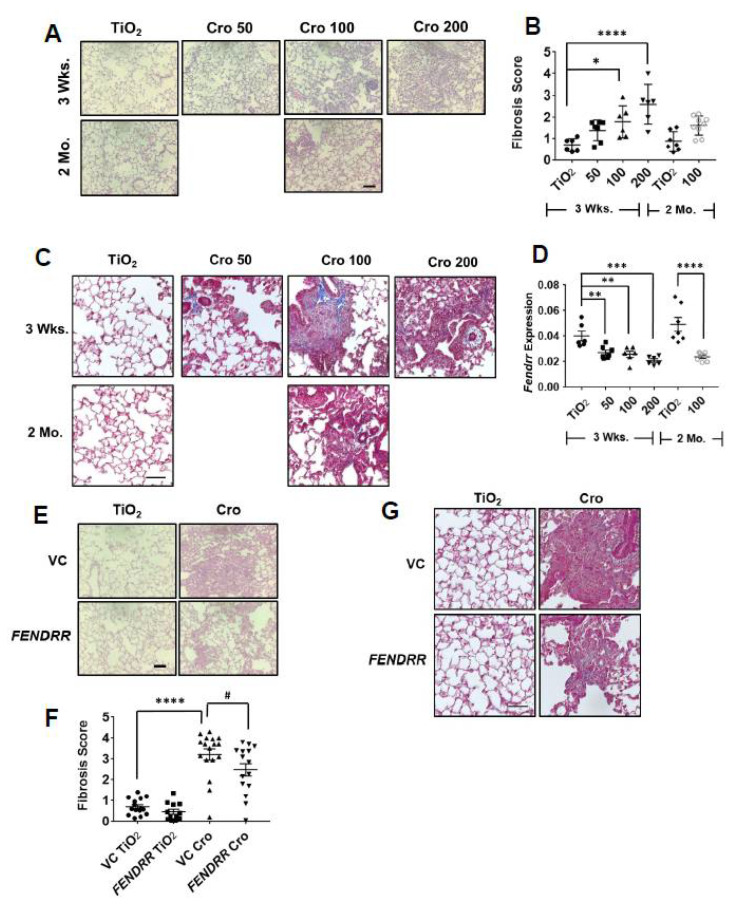
*FENDRR* reduces asbestos-induced lung fibrosis. (**A**) H & E staining of the lung tissues from the mice treated with different doses (50, 100, and 200 µg) of crocidolite asbestos (Cro) and collected at 3 weeks (wks) and 2 months (Mo). TiO_2_ (100 µg) was used as a control. (**B**) Fibrosis scores as determined by the modified Ashcroft score in the lungs of mice treated with crocidolite. Mouse number and sex for each group: 3 Wks, TiO_2_ = 6 (3F, 3M), Cro 50 = 7 (4F, 3M), Cro 100 = 6 (3F, 3M), Cro 200 = 6 (3F, 3M) and 2 Mo, TiO_2_ = 7 (4F, 3M), Cro 100 = 9 (6F, 3M). (**C**) Trichrome staining of lung sections of mice treated with crocidolite. (**D**) *Fendrr* expression of asbestos-treated mice as determined by real-time PCR using *Gapdh* as a control. Mouse number and sex for each group: 3 wks: TiO_2_ = 6 (3F, 3M), Cro 50 = 7 (4F, 3M), Cro 100 = 6 (3F, 3M), Cro 200 = 6 (3F, 3M) and 2 Mo, TiO_2_ = 7 (4F, 3M), Cro 100 = 9 (5F, 2M). (**E**) H & E staining of the lung sections from mice treated with virus control (VC) or *FENDRR* adenovirus and TiO_2_ or crocidolite (200 µg). (**F**) Fibrosis scores as determined by the modified Ashcroft score in the lungs of mice treated with *FENDRR* adenovirus and crocidolite. Mouse number and sex: VC TiO2 = 15 (8F, 7M), *FENDRR* TiO2 = 12 (6F, 6M), VC Cro = 17 (9F, 8M) and *FENDRR* Cro = 15 (7F, 8M). (**G**) Trichrome staining of the lung sections of mice treated with *FENDRR* adenovirus and crocidolite. Scale bar: 100 µm for all sections. Values represent the means ± SE. * *p* < 0.05, ** *p* < 0.01, *** *p* < 0.01, **** *p* < 0.0001, ^#^ *p* < 0.05. One-way ANOVA and Bonferroni’s multiple comparison for (**B**). One-way ANOVA and Fisher’s LSD test for (**D**,**F**).

We have previously shown that *FENDRR* reduces bleomycin-induced pulmonary fibrosis [15]. Although this model is commonly used, bleomycin-induced lung fibrosis is reversible at 4 weeks of the treatment. We thus tested the effects of adenovirus-mediated overexpression of *FENDRR* in the lung on asbestos-induced pulmonary fibrosis, which can persist for at least 2 months. Crocidolite asbestos (50, 100, and 200 µg/mouse) was intratracheally delivered into the lungs of C57BL/6J mice and lung fibrosis was evaluated at 3 weeks and 2 months. No mice died after asbestos delivery. H&E staining showed dose-dependent fibrotic lesions and increases in the fibrosis score, as determined by the modified Ashcroft scoring, at 3 weeks after asbestos delivery. Fibrosis was not resolved for up to 2 months (Figure 6A,B). This is consistent with collagen deposition as revealed by Mason’s trichrome staining, which shows blue color in fibrotic areas of mouse lungs (Figure 6C). Male and female mice showed a similar pattern of dose-dependent fibrosis scores at 3 weeks. However, at 2 months, the fibrosis score in the asbestos group was significantly different from controls in male but not in female mice, although there was a trend of increase in the fibrosis score in female groups (Figure S3A,B). Asbestos-treated mice showed a reduced *Fendrr* expression in the lungs at 3 weeks (32–48% reduction) and 2 months (51% reduction) after asbestos delivery (Figure 6D). There were no differences in male and female groups (Figure S3C,D). The treatment condition with 200 µg/mouse of crocidolite asbestos had dense fibrotic lesions and the highest fibrosis score and was chosen for further experiments.

Fibroblast proliferation is a key event in pulmonary fibrosis [25,26]. Since our in vitro studies showed that *FENDRR* inhibits fibroblast proliferation, we wondered whether *FENDRR* has an anti-fibrotic effect on asbestos-induced fibrosis. *FENDRR* was delivered to the lungs of mice using adenovirus-mediated gene transfer. We have previously shown that under the same conditions as used in this study, *FENDRR* expression was increased 1.7-fold after the delivery of an adenovirus expressing *FENDRR* into the lungs [15]. Adenovirus-mediated *FENDRR* transfer into the lungs reduced fibrotic lesions, fibrosis scores, and collagen deposition (Figure 6E–G). There were no apparent differences in fibrosis scores between male and female mice (Figure S3D,E).

### 2.7. RNA Sequencing Analysis Identifies Seven Cell Proliferation-Related Genes That Are Up-Regulated by Asbestos, but Attenuated by FENDRR

To evaluate the effect of *FENDDR* on the transcriptome in vivo, RNA sequencing was performed on the lung tissues from the asbestos-induced fibrosis mouse model with or without *FENDRR* overexpression. RNAs were extracted from lung tissues on day 21 from 4 groups of mice, vector control and crocidolite control (VC-TiO_2_), vector control and crocidolite (VC-Cro), *FENDRR* overexpression and crocidolite control (*FENDRR*-TiO_2_), and *FENDRR* overexpression and crocidolite (*FENDRR*-Cro), and RNA sequencing was performed as described in the Materials and Methods. The results revealed that a large number of genes (219 genes) were differentially expressed in the lungs of VC-Cro mice compared to VC-TiO_2_. Among them, 193 genes were up-regulated and 26 were down-regulated. Seventy genes (20 up-regulated and 50 down-regulated) were expressed differently between VC-Cro and *FENDRR*-Cro groups. The expression levels of 76 genes (14 up-regulated and 62 down-regulated) were different in the lungs of mice between VC-TiO_2_ and *FENDRR*-TiO_2_ (Figure S4A–C). Differentially expressed genes were visualized in volcano plots based on their fold changes and FDR. Red dots and green dots represent differentially up-regulated and down-regulated genes, respectively (FDR < 0.05 and fold change ≥ 2). Black dots indicate genes that did not change based on FDR ≥ 0.05 and fold change < 2 (Figure S4D–F).

The genes that were up-regulated in the VC-Cro group and down-regulated in the *FENDRR*-Cro group are listed in Table S1 Functional annotation on these genes showed that they are involved in collagen metabolic and catabolic processes, arginine metabolic and catabolic processes, immunity, and regulation of cell proliferation (Figure S5 and Table S2). The cellular components of these differentially expressed genes are involved in extracellular space (Figure S5 and Table S3). KEGG analysis showed that these genes are involved in arginine biosynthesis and metabolism (Figure S5 and Table S4).

Seven crocidolite-upregulated genes, which are attenuated by *FENDRR*, are related to cell proliferation (Tables S1 and S2). Those genes are arginase 1, glycoprotein Nmb, nitric oxide synthase 2, programmed cell death 1 ligand 2, scinderin, SWI/SNF related- Matrix associated-Actin dependent Regulator of Chromatin Subfamily A member 4, and Wnt Family Member 5A.

## 3. Discussion

**Figure 7:**
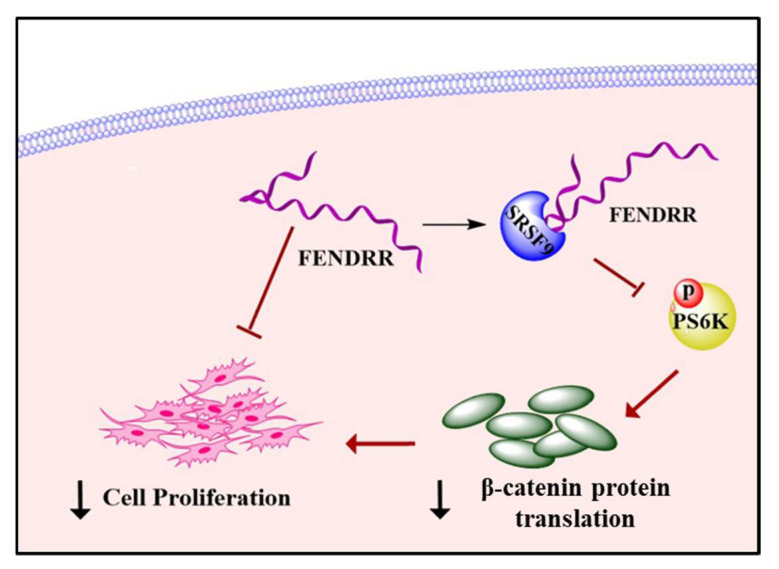
A model showing that FENDRR inhibits fibroblast proliferation by binding SRSF9 and reducing β-catenin protein levels.

The global incidence of IPF is rising annually [38] and the management of the disease has become important. The greatest challenge of disease management is that the current drug treatment options only slow down the disease progression, but cannot cure the disease [39,40]. Understanding key molecular mechanisms leading to the pathogenesis of IPF will help to develop effective treatment strategies. Fibroblast proliferation is a key characteristic of IPF [25,26,41,42]. Several studies have shown that *FENDRR* negatively affects cell proliferation in various cancers in the lungs [17,18,43,44], gastrointestinal tract [20,21] bone [45], and kidneys [19]. However, the exact cellular mechanisms on how *FENDRR* regulates cell proliferation are unclear. In this study, we demonstrate that *FENDRR* inhibits lung fibroblast proliferation. We also provide evidence to support a model that FENNDRR decreases fibroblast proliferation by binding SRSF9, inhibiting the phosphorylation of PS6K and reducing β-catenin protein level (Figure 7).

RNA immunoprecipitation analysis demonstrated that *FENDRR* bound SRSF9 (Figure 3A). The primary function of SRSF proteins is RNA splicing. However, the changes in the RS domains modulate the localization of these proteins in the nucleus or cytoplasm [32]. It has been shown that mutations in arginine sites to prevent methylation result in an increased shuttling of SRSF1 to the cytoplasm [46]. Also, mutations in RS domains that prevent the phosphorylation of RS domains increase the cytoplasmic accumulation of SRSF1 [47]. There is evidence showing that SRSF1 and SRSF7 in the cytosolic fraction are associated with ribosomal subunit, suggesting the involvement of SRSF proteins in mRNA translation [48]. Another study showed that hyper-phosphorylation of the RS domain in SRSF1 promoted the mRNA translation as determined by luciferase reporter assay [49]. Since SRSF9 can shuttle between the nucleus and the cytoplasm [32], and our previous results show that *FENDRR* is predominantly located in the cytoplasm in human lung fibroblasts, the binding of *FENDRR* with SRSF9 likely occurs in the cytoplasm.

SRSF9 is considered an oncogenic protein [23,33,34]. Our current studies reveal that the knockdown of *SRSF9* in lung fibroblasts inhibits cell proliferation, which is consistent with several studies in cancer cells. For example, silencing *SRSF9* in bladder cancer cells and neuroblastoma cells reduces cell proliferation [33,34] Subcutaneous tumor growth in mice is promoted by injecting NIH3T3 fibroblasts cells stably expressing SRSF9 [23].

The binding of *FENDRR* with SRSF9 could affect its downstream signaling. One such signaling is mTOR, which regulates cell metabolism, growth, proliferation, and survival. It enhances protein and lipid synthesis while reducing autophagy [50,51]. PS6K, one of the key components in mTOR signaling, regulates several proteins involved in protein translation. PS6K promotes translation initiation by phosphorylating eIF4B, which is a component of 5′-cap binding. PS6K also inactivates eukaryotic elongation factor 2 kinase (eEF2K), a negative regulator of eukaryotic elongation factor 2 (eEF2), and thus increases the translation elongation. Furthermore, PS6K enhances ribosome biogenesis by promoting the transcription of rRNA via phosphorylating the transcription factor UBF-1 [52,53].

The overexpression of SRSF1 and SRSF9 induces the phosphorylation of PS6K in HEK293T cells [23]. SRSF1 also activates mTOR signaling in MEF and NIH3T3 cells as determined by the phosphorylation of PS6K and 4E-BP [54]. In the lung fibroblasts stably expressing *FENDRR*, we observed increased phosphorylation of PS6K, suggesting that the binding of *FENDRR* with SRSF9 may inhibit mTOR signaling. The involvement of mTOR signaling in fibroblast proliferation is also supported by our finding that FGF2, a growth factor that activates mTOR signaling [55,56], induced the phosphorylation of PS6K and increased fibroblast proliferation.

The activation of mTOR signaling induces protein synthesis by translating genes responsible for cell growth and proliferation. β-catenin is one of the major mediators to promotes cell proliferation [28,29,57] and its synthesis is enhanced by SRSF1 and SRSF9 in an mTOR-dependent manner [23]. Thus, the *FENDRR*-mediated reduction in the phosphorylation of PS6K may potentially affect β-catenin protein levels and thus fibroblast proliferation. Indeed, *FENDRR* reduced the protein level, but not the mRNA level of β-catenin. Furthermore, reducing β-catenin levels in fibroblasts by gene silencing and chemical inhibition decreased cell proliferation. These results suggest that *FENDRR*-mediated inhibition of fibroblast proliferation is likely via a reduction in β-catenin protein level.

miR-214 is known to reduce the β-catenin expression in cancer cells [58,59,60]. We have previously shown that *FENDRR* competes with miR-214 [15]. This raises the possibility that *FENDRR* may regulate β-catenin protein levels via miR-214. However, this is not the case as our current studies showed that overexpression of *FENDRR* decreased β-catenin protein levels in lung fibroblasts. If *FENDRR*-mediated changes in β-catenin protein levels are via competing with miR-214, an increased β-catenin protein level should be observed in *FENDRR*-overexpressing cells. The plausible explanation is that β-catenin is likely not a target of miR-214 in lung fibroblasts since a microRNA-target relationship is cell context-dependent.

There are several lung fibrosis mouse models including bleomycin, paraquat, and asbestos [61]. Exposure to asbestos is considered a significant health hazard due to its potential risk of developing lung fibrosis and malignancies [62]. The alveolar epithelial injury caused by asbestos fiber-generated ROS is the hallmark to develop fibrosis in the lungs [41,63]. *FENDRR* was observed to be down-regulated in the lung of asbestos-treated mice. Although the precise mechanism by which asbestos reduces lung *FENDRR* expression is uncertain, we identified several potentially important pathways using RNA sequencing studies of *FENDRR*- and asbestos-treated mice. RNA sequencing identified that certain genes involved in the PPAR signaling pathway, chemokine signaling pathway, and IL-17 signaling pathway are up-regulated by crocidolite. These pathways may regulate *Fendrr* expression. However, further studies are required to explore the involvement of these pathways in fibrosis and *Fendrr* regulation. TGFβ1 signaling is another potential pathway to regulate *FENDRR* expression because TGFβ1 is increased in the lungs of asbestos-treated mice and rats [64] and is known to be involved in IPF. Our in vitro studies support the role of TGFβ1/Smad3 and hypoxia/HIF-1α signaling in the downregulation of *FENDRR* expression [15,65]. Adenovirus-mediated *FENDRR* transfer into the mouse lungs reduced asbestos-induced fibrotic lesions, fibrosis scores, and collagen deposition, suggesting that *FENDRR* is an anti-fibrotic lncRNA in vivo.

RNA sequencing identified 26 genes in the mouse lung tissues that were up-regulated by crocidolite but reversed by *FENDRR*. Among them, 7 genes [arginase 1 (*Arg1*), glycoprotein Nmb (*Gpnmb*), nitric oxide synthase 2 (*Nos2*), programmed cell death 1 ligand 2 (Pdcd1lg2), scinderin (*Scin*), SWI/SNF related, Matrix associated, Actin dependent Regulator of Chromatin, Subfamily A, member 4°(*Smarca4*), and Wnt Family Member 5A°(*Wnt5a*)] are reported to be involved in cell proliferation based on our functional annotation analysis.

Arginase is an enzyme important for the biosynthesis of polyamines from arginine. These polyamines are required to synthesize new cellular DNAs, RNAs, and proteins in the event of cell proliferation [66,67] including fibroblast proliferation [68]. Gpnmb is a type I transmembrane glycoprotein, and which is up-regulated in lung fibrosis mice models [69]. Furthermore, silencing of *GPNMB* has been shown to reduce the proliferation of osteosarcoma cells through suppressing mTOR signaling [70]. Nitirc oxide has been reported to enhance the Smad signaling to promote pulmonary fibrosis in rat models [71]. It was recently shown that cell surface expression of both PD-L1 and PD-L2 were increased in IPF fibroblasts compared to healthy controls [72], and the inhibition of Pd-l1 significantly reduced lung fibrosis in bleomycin-mice models [73]. SCIN is involved in cytoskeletal remodeling and the silencing of SCIN in cancer cells inhibits cell proliferation [74,75,76]. SMARCA4 is part of the chromatin remodeling complex SWI/SNF. SMARCA4 is highly expressed in human IPF lungs and the deletion of Smarca4 results in a decreased proliferation of alveolar type II cells [77]. WNT5a, a non-canonical Wnt ligand, is highly induced in lung fibrosis [78,79,80,81]. We have previously shown that WNT5a increases lung fibroblast proliferation through NFAT signaling [82]. Therefore, our RNA sequencing data provide new directions to further investigate molecular mechanisms associated with asbestos-induced lung fibrosis and *FENDRR* functions.

In summary, we conclude that *FENDRR* acts as an anti-fibrotic lncRNA to inhibit fibroblast proliferation by binding SRSF9 and inhibiting mTOR signaling, thereby reducing the β-catenin protein translation.

## 4. Materials and Methods

### 4.1. Cell Culture

Human lung fibroblasts, LL29 cells isolated from the lungs of an IPF patient were purchased from American Type Culture Collection (ATCC, Manassas, VA, USA). Primary human pulmonary fibroblasts (HPFs) isolated from the lungs of a healthy subject were purchased from PromoCell (Heidelberg, Germany, Cat. No: C-12361). LL29 cells were cultured in F12K medium with 10% fetal bovine serum and 1% penicillin-streptomycin. HPFs were cultured in fibroblast medium (PromoCell, Cat. No: C-23220) with its supplements (PromoCell, Cat. No: C-39320) containing fetal calf serum (0.2 mL/mL), basic fibroblast growth factor (1 ng/mL), and insulin (5 µg/mL).

### 4.2. Vector Construction and Virus Preparation

For the construction of a lentiviral human *FENDRR* expression vector, human lung tissue cDNA was used as a template to amplify *FENDRR* variant 3 (GeneBank ID: MK522493) by PCR. Primers used for the amplification are listed in Table S5. The PCR product and the pLVX/CMV-EGFP vector were double-digested to generate XhoI and EcoRI sticky ends. The digested PCR product was ligated to the pLVX/CMV-EGFP vector at XhoI and EcoRI sites. A random genomic DNA fragment (500 bp) was used to construct a control vector (VC), which did not contain any known sequence of mRNAs, lncRNAs, or microRNAs.

Lentiviral shRNA vectors for human *FENDRR* and *SRSF9* were constructed as previously described [83]. The sequences of shRNAs are listed in Table S5. A control vector (pMiRZip) was purchased from System Biosciences (Mountain View, CA, USA). Adenoviral β-catenin silencing vector containing 4 shRNAs and its control vector containing 4 non-relevant shRNAs [84] were constructed as previously described [85].

Lentiviruses and adenoviruses were prepared and titrated according to the previously reported methods [83,85].

### 4.3. Generation of Stable Cells Expressing FENDRR

To generate *FENDRR* and VC stable cells, LL29 cells were infected with a lentivirus expressing *FENDRR* or its control vector at a multiplicity of infection (MOI) of 50. After a 24-h of infection, the virus was removed, fresh medium was added, and cells were incubated for another 48 h. Cells were then cultured in the medium containing 0.5 µg/mL puromycin. The medium was replaced every 2 days until cells attained 70–80% confluence. Then, cells were sub-cultured and maintained in the medium containing 0.1 µg/mL of puromycin. During experimental conditions, these cells were cultured in the absence of puromycin.

### 4.4. RNA Isolation and Real-Time PCR

Tri reagent (Molecular Research Center, Cincinnati, OH, USA) was used to isolate total RNAs according to the manufacturer’s protocol. DNA digestion (ThermoFisher Scientific, Waltham, MA, USA) was performed, followed by cDNA synthesis using random primers. These cDNAs were diluted (1:100) and used for real-time PCR reaction as previously described [86]. The specificity and linearity of the qPCR assays were assessed by examining the melt curve for each primer pair and Ct values, respectively. Relative expression levels of mRNAs and lncRNA *FENDRR* were calculated using the comparative Ct method. β-actin or glyceraldehyde 3-phosphate dehydrogenase (GAPDH) was used as internal controls. Primers used for the real-time PCR were listed in Table S6.

### 4.5. RNA Immunoprecipitation (RIP)

LL29 cells were cultured in 10-cm dishes until a 70–80% confluence was attained. They were harvested with trypsinization and lysed with 1 mL of RIP buffer [50 mM KCl, 25 mM Tris (pH 7.4), 5 mM EDTA, and 0.5% NP-40] containing 1 U/µL RNase inhibitor (Super RNase inhibitor, Ambion, Foster City, CA, USA, Cat. No: AM2694), and 1x Halt^®^ protease inhibitor (ThermoFisher Scientific, Waltham, MA, USA, Cat. No:1861281). After centrifuging the lysate at 10,000× *g* for 15 min, the supernatant was collected and incubated with 40 µL of protein A/G beads (Santa Cruz Biotechnology, Santa Cruz, CA, USA, Cat. No: sc-2003) for 1 h at 4 °C. The mixture was centrifuged at 16,000× *g* for 15 min and the supernatant was collected. Fifty microliters were taken from this pre-cleared lysate and set aside as an input control. The remaining lysate was divided into two aliquots, and each aliquot was incubated with 10 µg of rabbit anti-SRSF9 antibody (Abcam, Boston, MA, USA, Cat. No 74782) or IgG control antibodies (Invitrogen, Waltham, MA, USA, Cat. No: 10500C) overnight at 4 °C. Forty microliters of protein A/G beads were added to each aliquot and incubated for 1 h at 4 °C. Beads were pelleted and washed with 500 µL of ice-cold RIP buffer three times, followed by 500 µL of a final ice-cold PBS wash. Beads were pelleted and PBS was removed completely. Then, TRI reagent (Molecular Research Center, Cincinnati, OH, USA) was added to isolate co-precipitated RNA. The RNAs were reverse-transcribed to cDNA. The amount of *FENDRR* in immunoprecipitated RNA was determined by real-time PCR and calculated using 2^−ct^. The enrichment fold was calculated over the IgG control.

### 4.6. Western Blot

Protein samples were extracted using a 1X SDS sample buffer containing 0.06 M Tris (pH 6.8), 2.1% (*w/v*) SDS, 5% (*v/v*) glycerol, and 1% (*v/v*) 2-mercapto-ethanol. Protein concentration was determined using a D_C_ protein assay kit (Bio-Rad, Hercules, CA, USA). Twelve µg of samples were separated on 10% SDS PAGE gels for detecting β-catenin, phospho-PS6K and PS6K, or 12% SDS PAGE gels for detecting SRSF9. Then, proteins were transferred to nitrocellulose membranes. Membranes were blocked with 5% milk for 1 h at room temperature. Primary antibodies used for Western blotting are as follows: rabbit anti-β-catenin (dilution 1:2000, Cell Signaling, Danvers, MA, USA, Cat. No: 9562), rabbit anti-SRSF9 (dilution 1:500, Abcam, Boston, MA Cat. No 74782), rabbit anti-PS6K (dilution 1:1,000, Cell Signaling, USA, Cat. No: 9202), rabbit anti-phosopho-PS6K (dilution 1:1000, Cell Signaling, USA, Cat. No: 9205), and mouse anti-β-actin (dilution 1:3000, ThermoFisher Scientific, Waltham, MA, USA, Cat. No: MA5-15739). The membranes were incubated with primary antibodies overnight at 4 °C. Then, membranes were incubated with horse-radish peroxidase-conjugated goat anti-rabbit or goat anti-mouse (dilution 1:2000) secondary antibodies (Jackson Immunoresearch, West Grove, PA, USA) for 1 h at room temperature. The PageRuler Prestained protein ladder (ThermoFisher Scientific, Waltham, MA, USA) was run along with samples in each blot. The signal was developed by adding chemiluminescent peroxidase substrate and images were taken with an Amersham Imager 600 (GE Healthcare, Pittsburg, PA). The protein band intensities were quantified using Image J software [87] (https://imagej.nih.gov, accessed on 1 August 2021) and normalized to an internal control, β-actin. The protein expression was represented as a percent of the control sample.

### 4.7. Nucleofection

Nucleofection reaction was carried out using Amaxa™ Basic Nucleofector™ Kit for Mammalian Fibroblasts (Lonza, Allendale, NJ, USA, Cat. No: VPI-1002). Briefly, 0.3 × 10^6^ cells in the nucleofection reagent were mixed with 2 µg of control vector (pGFP) or ΔGSK β-catenin overexpressing vector [43] kindly provided by Dr. Angela Barth from Stanford University), and the A-024 program was run with the Nucleofector II device (Lonza, Allendale, NJ, USA). Then, cells were suspended and cultured in F12K medium containing 10% fetal bovine serum and 1% penicillin-streptomycin.

### 4.8. Cell Proliferation Assay

Fibroblast proliferation was determined using a BrdU cell proliferation kit (EMD Millipore, St Charles, MO, USA). Otherwise indicated, 2000 cells/well were seeded in 96-well plates for all cell proliferation experiments. The following day the medium was replaced, and cells were infected with an adenovirus or a lentivirus at an MOI of 100. After 24-h of virus infection, the medium was replaced with fresh medium. Cells were then incubated for up to 6 days in F12K medium with serum. For chemical inhibition of β-catenin, cells were treated with 10 µM of XAV939 (Selleckchem, Houston, TX, USA) for 6 days. To activate the mTOR signaling, after 24 h serum starvation, cells were treated with Fibroblast Growth Factor (FGF) 2 at 50 ng/mL for 3 days in a serum-free medium. For all the experiments above, prior to the 12 h endpoint, the BrdU reagent was added and the assay was proceeded according to the manufacturer’s protocol.

### 4.9. A Mouse Model of Asbestos-Induced Pulmonary Fibrosis

All animal procedures used in this study were approved by the Institutional Animal Care and Use Committee at Oklahoma State University under the protocol number, VM 15–38. All experiments performed on mice were in accordance with the relevant guidelines and regulations of this committee. The study is reported in accordance with the ARRIVE guidelines. Asbestos preparation and delivery to mice were performed as previously described [88]. Crocidolite asbestos (kindly provided by Dr. Andy Ghio, US Environmental Protection Agency) and the control, TiO_2_ (Sigma-Aldrich, St Louis, MO, USA, Cat. No: 1667585) were dissolved in PBS containing 15 mM HEPES to obtain a stock concentration of 1, 2 or 4 mg/mL. The mixture was sonicated at 40% power for 8 min (Sonic Sonicator, Vibra Cell, Sonic & Materials Inc, Newtown, CT, USA, Cat. No: VCX 130PB). On day 1, male and female C57BL/6J mice (7–8 weeks old) were anesthetized using ketamine/xylazine. A 20-gauge Angiocath IV catheter was intubated in mice. Then, 50, 100, and 200 µg (in 50 µL) of crocidolite asbestos, or TiO_2_ were delivered intratracheally in two equal aliquots, two minutes apart. After each delivery, the mice were placed to the right and then left decubitus position for 10–15 s.

In another experiment, on day 0, male and female C57BL/6J mice (8 weeks old) were anesthetized using isoflurane and adenovirus expressing *FENDRR* or its vector control (VC) (50 µL) were intratracheally delivered at 5 × 10^9^ IU/mouse. On day 1, 200 µg of crocidolite or TiO_2_ was delivered as described above.

At 3 weeks or 2 months, mice were sacrificed by exsanguination under anesthesia using ketamine/xylazine. Then, lungs were perfused with sterile PBS via the right ventricle until they are pale in color. Left lungs were collected and snap-frozen using liquid nitrogen. The lung tissues were then powdered using a motor and pestle and lysed with Tri reagents, followed by RNA isolation. Right lungs were perfused with 10% formalin for histopathological analysis.

The degree of fibrosis was determined by the modified Ashcroft score [89]. Briefly, the slides were coded. Then, each of the 4 lobes of the right lung was imaged at a 20X objective lens magnification under a light microscope. Approximately, 7, 5, 5, and 3 fields were taken for inferior, superior, middle, and post cavel lobes, respectively, resulting in a total of 20 random fields per slide. A field that is covered by more than 50% of bronchi or vessels was excluded. Each image was blindly scored based on the modified Ascroft score as previously described [89], and an average score was obtained from 20 images per slide.

Collagen deposition in the mouse lungs were determined using Trichrome staining (Sigma-Aldrich, St. Louis, MO, USA).

### 4.10. Statistical Analysis

Values represent the means ± SE. Statistical analysis was performed using GraphPad Prism 7. We used the Student’s *t*-test for two-group comparison, a one-way ANOVA for multiple comparisons involving one factor or independent variable, and a two-way ANOVA for multiple comparisons involving two factors or independent variables, followed by Tukey’s or Bonferroni (if “n” is not equal among groups) or Fisher’s LSD post hoc test. A *p*-value of <0.05 was considered to be statistically significant.

## Data Availability

The RNA sequencing datasets are available at GEO (access number, GSE175496).

## Supplementary Materials

The following are available online at https://www.mdpi.com/article/10.3390/ijms22168536/s1, Supplementary Materials and Methods: RNAseq analysis. Figure S1: Silencing of SRSF9, Figure S2: Silencing of β-catenin, Figure S3: *FENDRR* reduced asbestos-induced lung fibrosis, Figure S4: Differentially expressed genes in the lung tissues of crocidolite- and *FENDRR*-treated mice, Figure S5: Functional annotation of crocidolite-up-regulated and *FENDRR*-down-regulated genes, Table S1: Crocidolite up-regulated and *FENDRR* down-regulated genes, Table S2: Biological processes of crocidolite up-regulated and *FENDRR* down-regulated genes, Table S3: Cellular components of crocidolite up-regulated and *FENDRR* down-regulated genes, Table S4: KEGG analysis of crocidolite up-regulated and *FENDRR* down-regulated genes. Table S5: Primers for the construction of plasmids, Table S6: Primers used for real-time PCR.
