# Supplementary material for: Long Noncoding RNA FENDRR Inhibits Lung Fibroblast Proliferation via a Reduction of β-Catenin
Source: Int J Mol Sci. 2021 Aug 9;22(16):8536. doi: 10.3390/ijms22168536 (PMC8395204; doi:10.3390/ijms22168536)
Supplement: Supplementary file 1 [file ijms-22-08536-s001.zip › ijms-1314427-supplementary.pdf]

Supplmentary Methods

RNA sequencing and data analysis

RNA sequencing was performed on the lung tissues collected on day 21 from *FENDRR* adenovirus- and/or asbestos-treated mice. RNAs were equally pooled (500 ng per animal) from each treatment group for each sex: VC-TiO_2 (male 7, female 8), *FENDRR*-TiO_2 (male 6, female 6), VC-Crocidolite (male 8, female 9) and *FENDRR*-Crocidolite (male 8, female 7). Therefore, 8 groups in total were generated for RNA sequencing. RNA quantitation and quality including RNA degradation, contamination, and integrity were assessed by using Nanodrop, Agilent Bioanalyzer 2100 and agarose gel electrophoresis. High-quality RNAs were used to prepare the sequencing library with the Illumina mRNA library preparation kit. In brief, mRNA was enriched using oligo(dT) beads. mRNA was fragmented. The first-strand cDNA was synthesized by using the mRNA template and random hexamers primer, followed by the second-strand cDNA synthesis using a second-strand synthesis buffer (Illumina), dNTPs, RNase H, and DNA polymerase I. A was added to 3′ end of the double-stranded cDNAs and then sequencing adaptor was ligated. Finally, the double-stranded cDNA library was subjected to size selection and PCR enrichment. The quality control of library was performed by the following three steps: (1) determining the library concentration preliminarily by Qubit 2.0, (2) assessing the insert size using Agilent 2100, and (3) quantifying the library effective concentration precisely via Q-PCR. The qualified libraries were sequenced using an Illumina sequencer. Each sample was sequenced to generate a minimum of 20 million reads. The sequenced reads were filtered by removing the reads containing (1) adapters, (2) more than 10% of the bases that can not be determined or (3) more than 50% of the bases that are low quality (Qscore<= 5) base.

The clean paired-end reads were directionally mapped to the mouse genome (GRCm38.p6) by TopHat2 with the following parameters: Num-threads were set to 24, GTF-formatted file containing mRNA gene annotation was supplied, transcriptome-index was set to transcriptome data/known, the coverage-based search for junctions was disabled, splice-mismatches was set to 1, and min-anchor-length was set to 7.

Cufflink and CuffDiff analysis for mixed genders (4 treatment groups) were run to identify the differentially expressed genes in these samples with the following parameters: Num-threads was set to 12, GTF-formatted file containing mRNA gene annotation was supplied, and a false discovery rate (FDR) value was 0.05. Genes with a fold change of ≥ 2 and FDR of < 0.05 were considered to be differentially expressed. The function annotation was performed by using STRING analysis (https://string-db.org/). The RNA sequencing datasets have been submitted to GEO (access number, GSE175496).

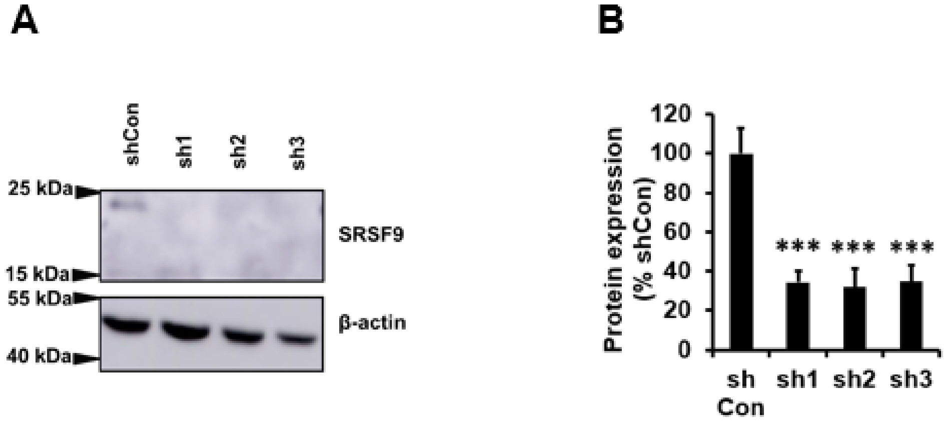

Figure S1. **Silencing of SRSF9.** (**A**) Western blot showing the silencing of SRSF9 protein in LL29 cells by a lentiviral shRNA (MOI 100 for 24 hrs) for SRSF9 (sh1, sh2 and sh3). shCon: vector control. (**B**) Quantitative analysis of the silencing efficiency of the SRSF9 shRNA at the protein level. The results were normalized to β-actin and expressed as %shCon. Values represent means ± SE. n=3 independent experiments. ***P<0.001 vs shCon. One-way ANOVA and Tukey's multiple comparison.

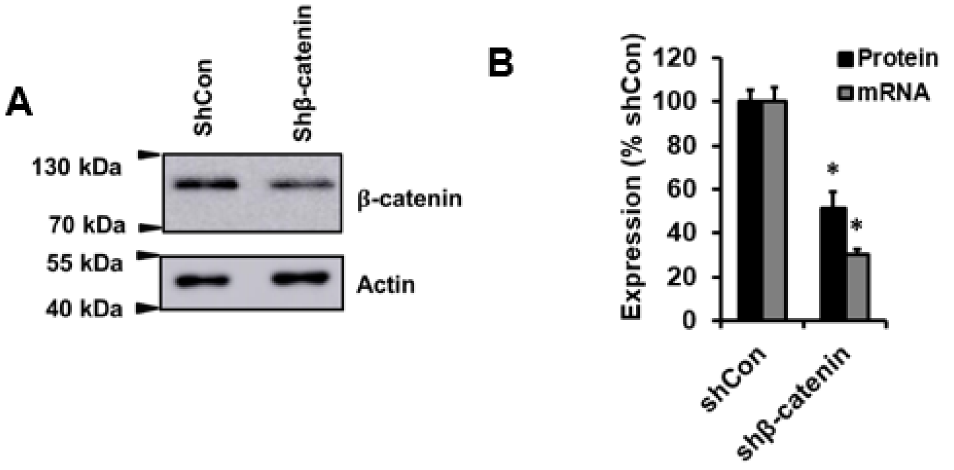

Figure S2. Silencing of β-catenin**.** (**A**) Western blot showing adenoviral shRNA-mediated silencing (MOI 100 for 24 hrs) of β-catenin protein levels in LL29 cells. shCon: shRNA control. (**B**) Quantitative analysis of β-catenin mRNA and protein levels after adenoviral shRNA-mediated silencing in LL29 cells. The results were normalized to β-actin and expressed as %shCon. Values represent means ± SE. n=3 independent experiments. *P < 0.05, vs shCon.. Student's *t-test*.

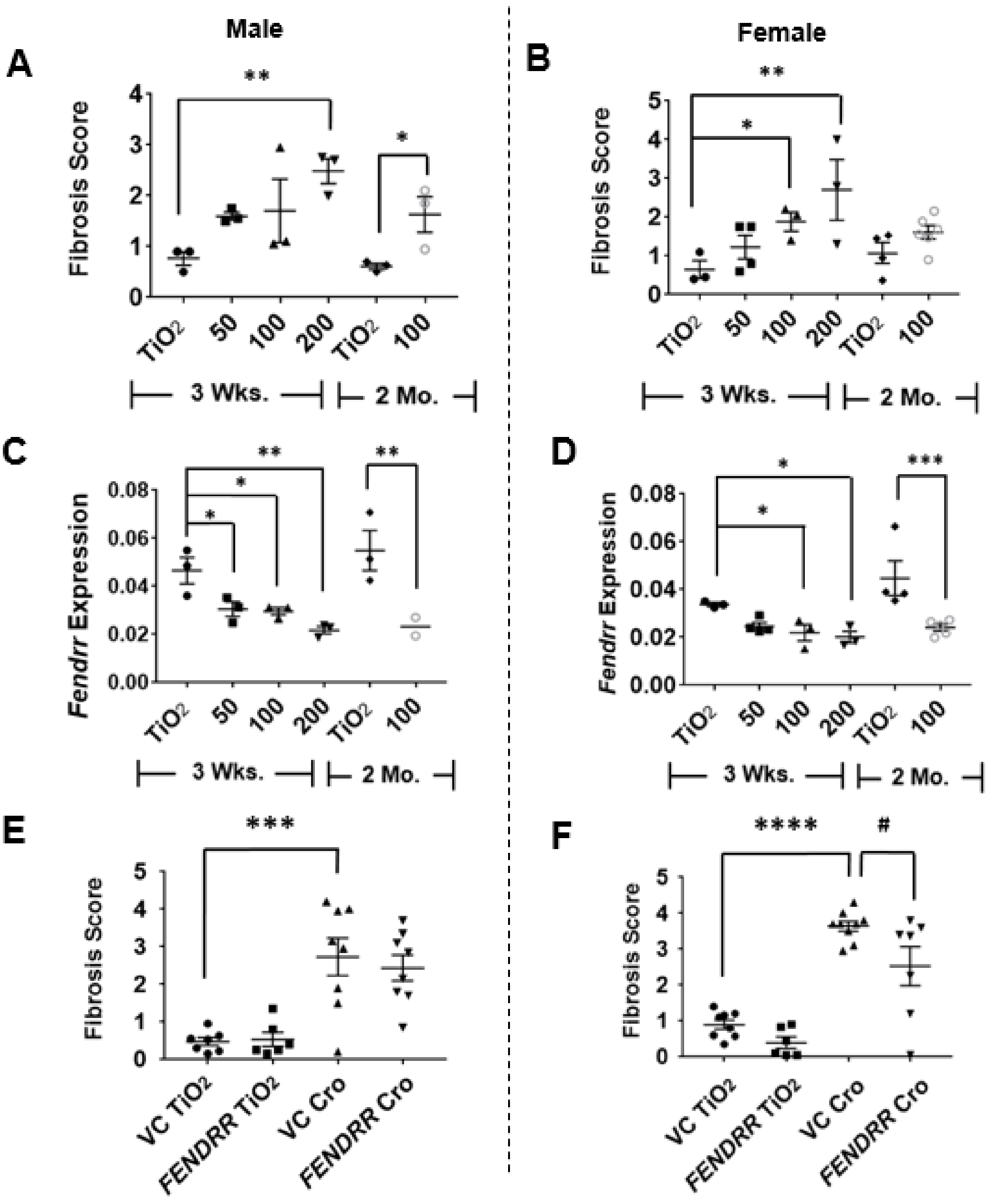

Figure S3. *FENDRR* reduced asbestos-induced lung fibrosis**.** Fibrosis scores and *Fendrr* expression of (**A, C**) male and (**B, D**) female mice exposed to different doses of crocidolite (Cro, μg per mouse) or control (TiO_2, 100 μg per mouse) for 3 weeks (wks) or 2 months (Mo). Fibrosis score of **(E)** male and **(F)** female mice treated with *FENDRR*, or virus control (VC) and crocidolite or TiO_2. Each symbol represent one animal. *P<0.05, **P<0.01, ***P<0.001, ****P<0.0001. #P<0.05 and #P<0.01. One-way ANOVA and Fisher's LSD test was performed for multiple comparison for A-D and One-way ANOVA and Bonferroni's multiple comparison was performed for E and F.

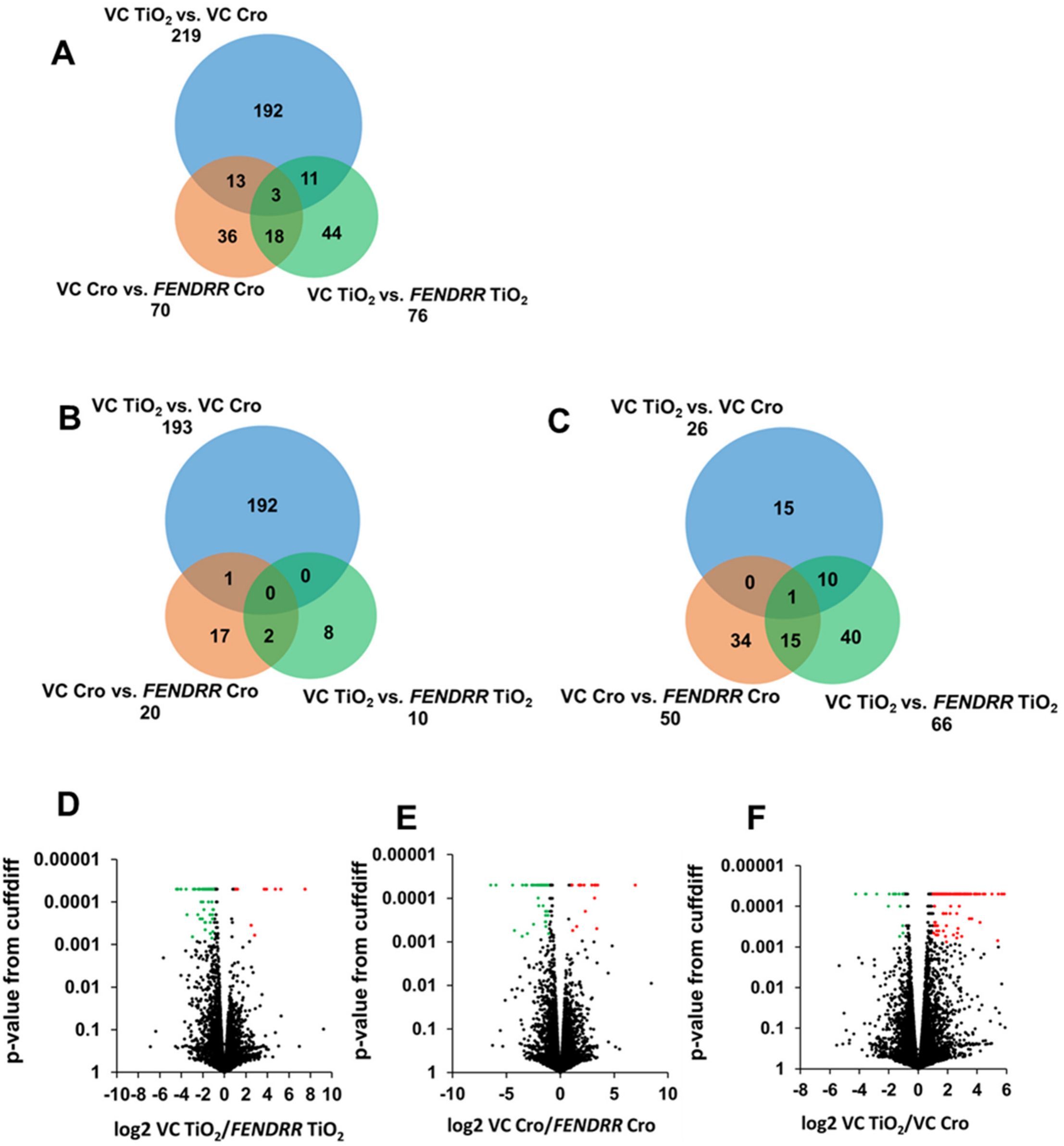

Figure S4. Differentially expressed genes in the lung tissues of crocidolite- and *FENDRR*-treated mice. Venn diagrams showing (**A**) total changed, (**B**) up-regulated, (**C**) down-regulated genes among different groups (FDR < 0.05 and fold change ≥2). The number of changed genes between two comparison groups is shown under treatment groups. The number in each cycle represents the number of changed genes between two comparison groups and the numbers in overlapping cycles represent the numbers of common changed genes among the comparison groups. (**D-F**) Volcano plots showing the distribution of differentially expressed genes between VC-TiO2 and *FENDRR*-TiO_2, VC-Cro and *FENDRR*-Cro, and VC-TiO2 and VC-Cro. Red dots indicate significantly up-regulated transcripts and green dots indicate significantly down-regulated transcripts (FDR < 0.05 and fold change ≥2). Black dots indicate unchanged (fold change below 2) and non-significant transcripts (FDR ≥ 0.05).

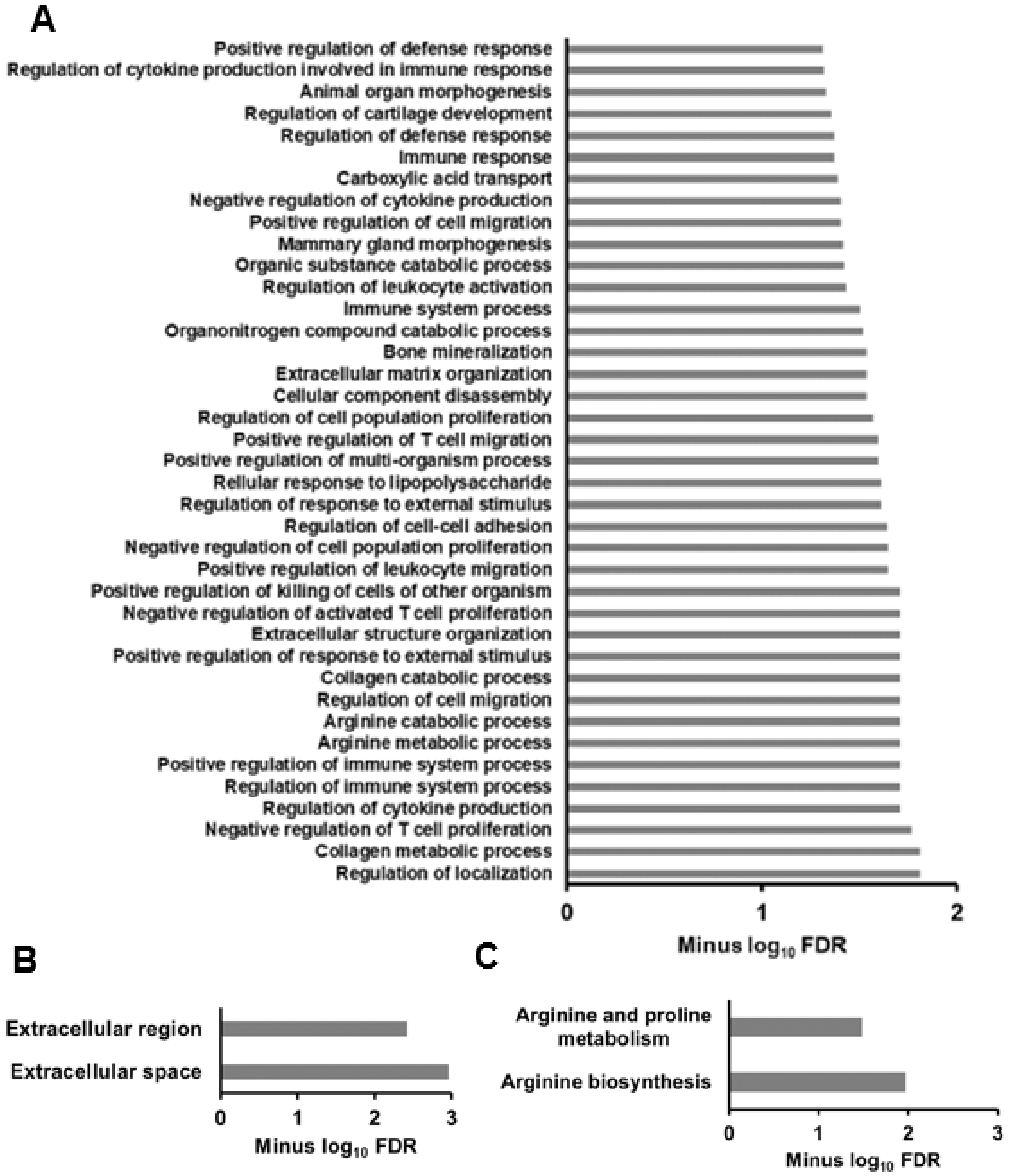

Figure S5. Functional annotation of crocidolite-up-regulated and *FENDRR*-down-regulated genes. (**A**) Biological processes. (**B**) Cellular components. (**C**) KEGG analysis. FDR were represented as minus $\log_{10}$ FDR.

Table S1. Crocidolite up-regulated and *FENDRR* down-regulated genes.

Gene Abbreviation	Gene Name	FPKM		
		VC-TiO_2	VC-Cro	FENDRR-

				Cro
Phf3	PHD Finger Protein 3	21.2	44	20
Tmem26	Transmembrane Protein 26	0.3	1.9	0.8
Arg1	Arginase 1	2.3	118.5	62.2
Gas2l3	Growth Arrest Specific 2 Like 3	0.6	3.5	0.8
Nos2	Nitric Oxide Synthase 2	1.9	4.5	2.4
Serpina3g	Serpin Family A Member 3	30.9	64.6	37
Slc26a4	Solute Carrier Family 26 Member 4	5.9	89.5	54
Scin	Scinderin	0.5	5.5	3.1
Wnt5a	Wnt Family Member 5A	3.7	8.2	3.6
*RP23-378M19.1**	N/A	0	1.6	0
Tbccd1	TBCC Domain Containing 1	6.1	16.1	6.5
Pla2g7	Phospholipase A2 Group VII	8.8	30	17.8
H2-M2	Histocompatibility 2, M region locus 2	4.7	28.3	16.5
Pdcd1lg2	Programmed Cell Death 1 Ligand 2	0.8	5.3	2.5
RP23-401J24.3*, RP23-401J24.4*	N/A	1.4	16.4	1.9
Ctss	Cathepsin S	410.9	945.9	557.2
Chil4	Chitinase-like protein 4	0.3	346.7	189.2
*RP23-360J20.1**	N/A	0.8	8.2	1.5
Slc15a4	Solute Carrier Family 15 Member 4	28.9	87.4	14.6
Gpnmb	Glycoprotein Nmb	15.3	116.8	76
Igkv5-43	Immunoglobulin kappa chain variable 5-43	38.8	108	17.4
Slc5a11	Solute Carrier Family 5 Member 11	0.1	7.6	0.09
Mmp13	Matrix Metallopeptidase 13	0.8	7.8	4.1
Smarca4	SWI/SNF Related, Matrix Associated, Actin Dependent Regulator Of Chromatin, Subfamily A, Member 4	21.6	55.6	21
Cd99l2	CD99 Molecule Like 2	18.6	49.5	15.8

* Gene name is not available (N/A)

Table S2: Biological processes of crocidolite up-regulated and FENDRR down-regulated genes

**Term ID**	**Term description**	**Observed gene [illegible]**	**Background gene**	**FDR**	**Matching genes in network**
GO:0032879	Regulation of localization	11	2579	0.0156	*Arg1,Cd99l2,Ctss,Gpnmb,Mmp13,Nos2,Pla2g7,Slc26a4,Smarca4,Tbccd1,Wnt5a*
GO:0032963	Collagen metabolic process	3	40	0.0156	*Arg1,Ctss,Mmp13*
GO:0042130	Negative regulation of T cell proliferation	3	61	0.0173	*Arg1,Gpnmb,Pdcd1lg2*
GO:0001817	Regulation of cytokine production	5	592	0.0196	*Arg1,Gpnmb,Nos2,Pdcd1lg2,Wnt5a*
GO:0002682	Regulation of immune system process	7	1165	0.0196	*Arg1,Cd99l2,Gpnmb,Pdcd1lg2,Pla2g7,Scin,Wnt5a*
GO:0002684	Positive regulation of immune system process	6	771	0.0196	*Arg1,Cd99l2,Pdcd1lg2,Pla2g7,Scin,Wnt5a*
GO:0006525	Arginine metabolic process	2	16	0.0196	*Arg1,Nos2*
GO:0006527	Arginine catabolic process	2	9	0.0196	*Arg1,Nos2*
GO:0030334	Regulation of cell migration	6	805	0.0196	*Cd99l2,Gpnmb,Pla2g7,Smarca4,Tbccd1,Wnt5a*
GO:0030574	Collagen catabolic process	2	22	0.0196	*Ctss,Mmp13*
GO:0032103	Positive regulation of response to external stimulus	4	285	0.0196	*Arg1,Ctss,Pla2g7,Wnt5a*
GO:0043062	Extracellular structure organization	4	214	0.0196	*Ctss,Mmp13,Pla2g7,Smarca4*
GO:0046007	Negative regulation of activated T cell proliferation	2	9	0.0196	*Arg1,Pdcd1lg2*
GO:0051712	Positive regulation of killing of cells of other organism	2	14	0.0196	*Arg1,Nos2*
GO:0002687	Positive regulation of leukocyte migration	3	143	0.0225	*Cd99l2,Pla2g7,Wnt5a*
GO:0008285	Negative regulation of cell population proliferation	5	648	0.0225	*Arg1,Gpnmb,Pdcd1lg2,Scin,Wnt5a*
GO:0022407	Regulation of cell-cell adhesion	4	360	0.0227	*Arg1,Gpnmb,Pdcd1lg2,Wnt5a*
GO:0032101	Regulation of response to external stimulus	5	681	0.0247	*Arg1,Ctss,Nos2,Pla2g7,Wnt5a*
GO:0071222	Cellular response to lipopolysaccharide	3	152	0.0247	*Arg1,Nos2,Wnt5a*

GO:0043902	Positive regulation of multi-organism process	3	158	0.0254	*Arg1,Nos2,Smarca4*
GO:2000406	Positive regulation of T cell migration	2	32	0.0254	*Cd99l2,Wnt5a*
GO:0042127	Regulation of cell population proliferation	7	1594	0.0268	*Arg1,Gpnmb,Nos2,Pdcd1lg2,Scin,Smarca4,Wnt5a*
GO:0022411	Cellular component disassembly	3	185	0.029	*Ctss,Mmp13,Smarca4*
GO:0030198	Extracellular matrix organization	3	180	0.029	*Ctss,Mmp13,Smarca4*
GO:0030282	Bone mineralization	2	40	0.029	*Gpnmb,Mmp13*
GO:1901565	Organo-nitrogen compound catabolic process	5	790	0.0304	*Arg1,Chil4,Ctss,Mmp13,Nos2*
GO:0002376	Immune system process	7	1703	0.0314	*Arg1,Cd99l2,Ctss,Nos2,Serpina3g,Smarca4,Wnt5a*
GO:0002694	Regulation of leukocyte activation	4	479	0.0373	*Arg1,Gpnmb,Pdcd1lg2,Wnt5a*
GO:1901575	Organic substance catabolic process	6	1276	0.0382	*Arg1,Chil4,Ctss,Mmp13,Nos2,Pla2g7*
GO:0060443	Mammary gland morphogenesis	2	53	0.0388	*Arg1,Wnt5a*
GO:0030335	Positive regulation of cell migration	4	500	0.0395	*Cd99l2,Gpnmb,Pla2g7,Wnt5a*
GO:0001818	Negative regulation of cytokine production	3	229	0.0396	*Arg1,Gpnmb,Pdcd1lg2*
GO:0046942	Carboxylic acid transport	3	233	0.0409	*Nos2,Slc15a4,Slc26a4*
GO:0006955	Immune response	5	914	0.0429	*Arg1,Ctss,Nos2,Serpina3g,Wnt5a*
GO:0031347	Regulation of defense response	4	538	0.0429	*Arg1,Ctss,Nos2,Wnt5a*
GO:0061035	Regulation of cartilage development	2	64	0.0441	*Scin,Wnt5a*
GO:0009887	Animal organ morphogenesis	5	956	0.0473	*Arg1,Mmp13,Slc26a4,Smarca4,Wnt5a*
GO:0002718	Regulation of cytokine production involved in immune response	2	69	0.0481	*Arg1,Wnt5a*
GO:0031349	Positive regulation of defense response	3	268	0.049	*Arg1,Ctss,Wnt5a*

*Number of significantly changed genes in the input which are involved in a GO term. # Total number of genes annotated to a GO term

Table S3: Cellular components of crocidolite up-regulated and FENDRR down-regulated genes

Term ID	Term description	Observed gene count*	Background gene count#	FDR	Matching genes in network
GO:0005615	Extracellular space	8	1131	0.0011	*Arg1, Ctss, Mmp13, Nos2, Pla2g7, Serpina3g, Slc26a4, Wnt5a*
GO:0005576	Extracellular region	9	2044	0.0038	*Arg1, Chil4, Ctss, Mmp13, Nos2, Pla2g7, Serpina3g, Slc26a4, Wnt5a*

*Number of significantly changed genes in the input which are involved in a GO term

Total number of genes annotated to a GO term

Table S4: KEGG analysis of crocidolite up-regulated and *FENDRR* down-regulated genes

Term ID	Term description	Observed gene count*	Background gene count#	FDR	Matching genes in network
mmu00220	Arginine biosynthesis	2	19	0.0106	*Arg1, Nos2*
mmu00330	Arginine and proline metabolism	2	50	0.0329	*Arg1, Nos2*

*Number of significantly changed genes in the input which are involved in a GO term

Total number of genes annotated to a GO term

Table S5: Primers for the construction of plasmids

FENDRR-FW	TTTCTCGAGCAGACAGCGCGGGCTGGGAG
FENDRR-RE	TTTGGTCTCGAATTGTCCATCGAGTTGTCATGCTT
FENDRR-shRNA-FW	GATCCGATTTGCCAGCAACTGCATCATTCAAGAGATGATGCA GTTGCTGGCAAATCTTTTTG
FENDRR-shRNA-RE	AATTCAAAAAGATTTGCCAGCAACTGCATCATCTCTTGAATG ATGCAGTTGCTGGCAAATCG
SRSF9-shRNA-FW1	GATCCGGAATATGCCCTGCGTAAACTTTCAAGAGAAGTTTAC GCAGGGCATATTCCTTTTTG
SRSF9-shRNA-RE1	AATTCAAAAAGGAATATGCCCTGCGTAAACTTCTCTTGAAAG TTTACGCAGGGCATATTCCG
SRSF9-shRNA-FW2	GATCCGCAGAGGATGCTATTTATGGATTCAAGAGATCCATAA ATAGCATCCTCTGCTTTTTG
SRSF9-shRNA-RE2	AATTCAAAAAGCAGAGGATGCTATTTATGGATCTCTTGAATC CATAAATAGCATCCTCTGCG
SRSF9-shRNA-FW3	GATCCGGAGGACCTGTTCTACAAGTATTCAAGAGATACTTGT AGAACAGGTCCTCCTTTTTG
SRSF9-shRNA-RE3	AATTCAAAAAGGAGGACCTGTTCTACAAGTATCTCTTGAATA CTTGTAGAACAGGTCCTCCG

FW: Forward, RE: Reverse. Species: human

Table S6: Primers used for real-time PCR

h*TCF1*-FW	AGGCCAAGAAGCCAACCATCAAGA
h*TCF1*-RE	ACTCTGCAATGACCTTGGCTCTCA
h*LEF1*-FW	GCTTTATCCAGGCTGGTCTGCAA
h*LEF1*-RE	GACCTGTACCTGATGCAGATTCCT
h*AXIN2*-FW	ACAACAGCATTGTCTCCAAGCAGC
h*AXIN2*-RE	GCGCCTGGTCAAACATGATGGAAT
h*FENDRR*-FW	GCGCACAGACCCAGGATTT
h*FENDRR*-RE	CACGGGCAGAGCTGGTTT
hβ-actin-FW	GCCGGGACCTGACTGACTAC
hβ-actin-RE	TTCTCCTTAATGTCACGCACGAT
m*Fendrr*-FW	CACGATCCCAGGTGGACTTG
m*Fendrr*-RE	TGCAGGAGTGAAGGGTGTCTCT
m*Gapdh*-FW	CTCGTCCCGTAGACAAAATGGT
m*Gapdh*-RE	TGATGGCAACAATCTCCACTT

FW: Forward, RE: reverse

"h" stands for human and "m" stands for mouse
